# Gap junction-mediated regulation of endothelial cellular stiffness

**DOI:** 10.1038/s41598-017-06463-x

**Published:** 2017-07-21

**Authors:** Takayuki Okamoto, Eiji Kawamoto, Yoshimi Takagi, Nobuyuki Akita, Tatsuya Hayashi, Eun Jeong Park, Koji Suzuki, Motomu Shimaoka

**Affiliations:** 1 Department of Pharmacology, Faculty of Medicine, Shimane University, 89-1 Enya-cho, Izumo-city, Shimane 693-8501 Japan; 2 Department of Molecular Pathobiology and Cell Adhesion Biology, Mie University Graduate School of Medicine, 2-174 Edobashi, Tsu-city, Mie 514-8507 Japan; 3 Emergency and Critical Care Center, Mie University Hospital, 2-174 Edobashi, Tsu-city, 514-8507 Japan; 4 Faculty of Medical Engineering, Suzuka University of Medical Science, 1001-1, Kishioka-cho, Suzuka-city, Mie 510-0293 Japan; 5 Department of Biochemistry, Mie Prefectural College of Nursing, 1-1-1 Yumegaoka, Tsu-city, Mie 514-0116 Japan; 6 Faculty of Pharmaceutical Science, Suzuka University of Medical Science, 3500-3, Minamitamagaki-cho, Suzuka-city, Mie 513-8679 Japan

## Abstract

Endothelial monolayers have shown the ability to signal each other through gap junctions. Gap junction-mediated cell-cell interactions have been implicated in the modulation of endothelial cell functions during vascular inflammation. Inflammatory mediators alter the mechanical properties of endothelial cells, although the exact role of gap junctions in this process remains unclear. Here, we sought to study the role of gap junctions in the regulation of endothelial stiffness, an important physical feature that is associated with many vascular pathologies. The endothelial cellular stiffness of living endothelial cells was determined by using atomic force microscopy. We found that tumor necrosis factor-α transiently increased endothelial cellular stiffness, which is regulated by cytoskeletal rearrangement and cell-cell interactions. We explored the role of gap junctions in endothelial cellular stiffening by utilizing gap junction blockers, carbenoxolone, inhibitory anti-connexin 32 antibody or anti-connexin 43 antibody. Blockade of gap junctions induced the cellular stiffening associated with focal adhesion formation and cytoskeletal rearrangement, and prolonged tumor necrosis factor-α-induced endothelial cellular stiffening. These results suggest that gap junction-mediated cell-cell interactions play an important role in the regulation of endothelial cellular stiffness.

## Introduction

Endothelial cells (ECs) have been shown *in vitro* to increase their cellular stiffness when they were subjected to shear stress^1^, pro-inflammatory cytokine tumor necrosis factor-α (TNF-α)^2^, and oxidized low-density lipoprotein^3, 4^. Depletion of cholesterol was found to increase endothelial cellular stiffness^5^. Furthermore, sub-endothelial substrate stiffness has been shown to be an important determinant of endothelial cellular stiffness^6, 7^. Increased stiffness of the vascular wall and sub-endothelial tissues has been implicated in the pathogenesis of atherosclerosis^8, 9^ and vascular inflammation^10, 11^; however, mechanisms regulating alteration of ECs themselves remains less well defined.

Cytoskeletal rearrangement plays a major role in the regulation of cellular stiffness^9, 12^, and both the actomyosin cytoskeleton and filamentous actin (F-actin) are important determinants of cellular stiffness^13^. The Rho-actomyosin pathway is known to be involved in regulating the cytoskeletal rearrangement induced by inflammatory mediators such as thrombin^14^ and TNF-α^15^. Rho kinase inhibits myosin light chain phosphatase, promoting the phosphorylation of myosin light chains and resulting in increased myosin activity in the actomyosin cytoskeleton^16, 17^. Activation of the Rho-actomyosin signaling pathway enhances the formation of actin bundles, stress fibers, and tensile actomyosin structures^18^, all of which correlate with cellular stiffness^13, 19^. The Rho pathway is also involved in integrin-dependent focal adhesion formation^20^. Integrins are essential for sensing substrate rigidity and generating the contractile forces associated with actin rearrangement^21^ that lead to cellular stiffening. This suggests the important roles played by integrin-mediated EC-extracellular matrix interactions in regulating endothelial cellular stiffness.

In addition to the interaction of ECs with the sub-endothelial matrix, the lateral hemophilic interactions between ECs have similarly been suggested to play a role in regulating cellular stiffness^12, 22^. Whereas gap junctions (GJs) are formed between ECs, the specific contributions of GJs in regulating cellular stiffness have yet to be elucidated. GJs connect and synchronize the intracellular environment of neighboring cells by promoting the transfer of ions, amino acids, small metabolites, and secondary messengers^23, 24^. GJs are formed by members of the connexin (Cx) family, which contains at least 20 highly conserved proteins with tissue-specific expression patterns^25^. Cx32, Cx37, Cx40, and Cx43 are expressed by ECs^26, 27^. These Cxs induce signaling via associating proteins, such as regulatory proteins, phosphatases and protein kinases, catenins, structural proteins, and microtubules^28^. Deletion of Cx40 from ECs, as well as the dysfunction of Cx37, can promote the development of atherosclerosis by enhancing both monocyte adhesion and transmigration^29, 30^. Alternatively, reduced expression of Cx43 by smooth muscle cells inhibits the formation of atherosclerotic lesions^31^, while the deletion of Cx43 modulates renin secretion, thereby leading to hypertension^32^. A Cx43 mutation in patients with cardiac infarction has been identified^33^.

Abnormal expression and dysfunction of endothelial Cxs have been associated with the onset of cardiovascular diseases^29–31^. We have previously shown that the Cx32-mediated intercellular transfer of small molecules decreases upon inflammation^34^, and that aberrant endothelial Cx32 increases pro-inflammatory cytokines^34^ and pro-coagulant tissue factor expression^35^. Furthermore, we have shown that Cx32 enhances angiogenesis-related endothelial tube formation and migration, while Cx43 reduces them^36^. Although EC-EC communications via Cxs have been shown to regulate many EC functions such as leukocyte adhesion^29, 30^, vascular permeability^37^, and angiogenesis^38^, it remains to be determined how Cxs regulate cellular stiffness. Here, using an atomic force microscopy (AFM)-based approach, we aimed to investigate whether endothelial cellular stiffening is induced by the aberrant regulation of GJs caused by inflammatory mediators.

## Results

### ECs stiffness increases in response to inflammatory stimulation

**Figure 1:**
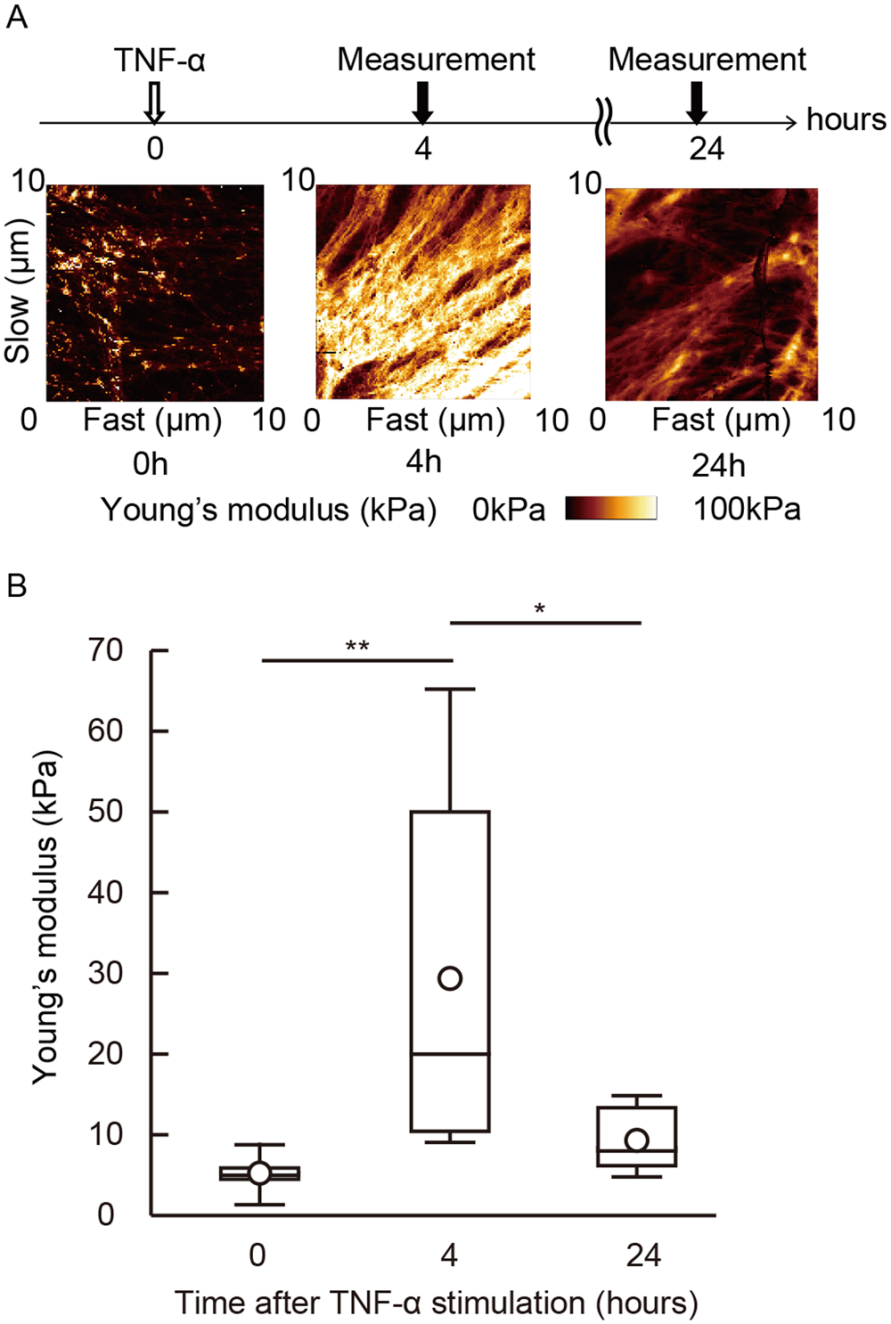
EC increased its stiffness in response to TNF-α stimulation. Cellular stiffness of the cell body was determined using AFM. (**A**) HUVECs were stimulated with TNF-α and force curves of living cells were measured at the indicated time points. Young’s modulus images were reconstructed. (**B**) Young’s modulus of HUVECs was determined after TNF-α stimulation. The geometric mean of Young’s modulus of HUVECs at 0 (n = 7), 4 (n = 6), and 24 hours (n = 7) are shown. Representative data from three experiments are shown. *P < 0.05, **P < 0.01; measured using a Tukey’s test followed by two-way ANOVA.

In order to determine the stiffness of primary human umbilical vein endothelial cells (HUVECs), we utilized AFM to measure the force curve only on the cell body, excluding the measurements on the nucleus or the cell edge. Cell stiffness was determined by analyzing the obtained force curve and reconstructing it as a stiffness image. The mean stiffness of normal HUVECs was approximately 10.4 kPa. In addition, we measured the stiffness of human aortic ECs (8.2 kPa), human pulmonary artery ECs (7.8 kPa), and human lung microvascular ECs (9.5 kPa). To study endothelial cellular stiffness upon stimulation with inflammatory mediators, we used HUVECs treated with the pro-inflammatory cytokine TNF-α, in order to model inflamed endothelial cells^39^. We stimulated confluent HUVECs with TNF-α for 4 hours and 24 hours in serum-free media, and then measured cellular stiffness (Fig. 1A). After 4 hours, endothelial cellular stiffness was remarkably increased, compared to non-stimulated control cells; rigid fibrillate structures were observed in TNF-α-stimulated HUVECs (Fig. 1A). Although ECs increased their stiffness in response to TNF-α stimulation at 4 hours, the stiffness of HUVECs returned to baseline levels at 24 hours.

Thrombin, another inflammatory mediator that processes pro-coagulation activity, was tested. Thrombin activates ECs via protease activated receptor-1 and then induced Rho-mediated contraction^14^. Subsequently, Rho induced an increase in vascular permeability and the inflammatory responses associated with actin rearrangement^14^. Thrombin stimulation increased cellular stiffness after stimulation for 4 hours, and returned to baseline levels at 24 hours, as is shown in the stimulatory conditions using TNF-α (Supplemental Fig. 1). These results have shown that ECs increase cellular stiffness in response to pro-inflammatory stimulation.

### Actin rearrangement aids the increase of endothelial cellular stiffness

**Figure 2:**
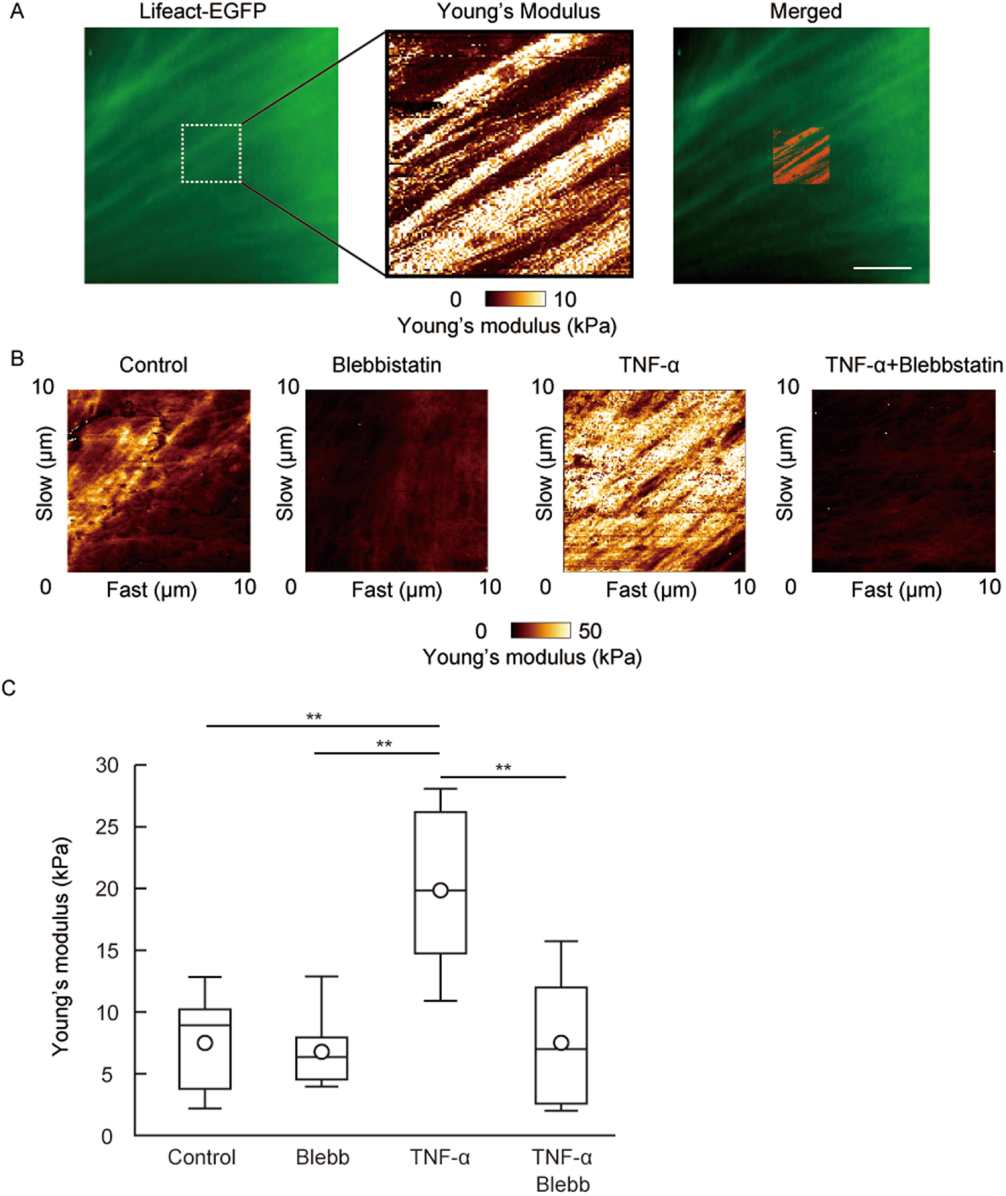
Actin localization at the area of stiffness in the cell. (**A**) Actin localization in living HUVECs was visualized by Lifeact-EGFP and the cellular stiffness of the actin-localized part of HUVECs was measured (n = 3). Actin localization (left panel) and Young’s modulus image (center panel) are shown. Both images of actin localization and the Young’s modulus were merged (right panel). Scale bar showing 10 μm. (**B**) Young’s modulus images of HUVECs treated with 10 μM blebbistatin and/or TNF-α for 4 hours are shown. (**C**) The Young’s modulus of control group (n = 5), blebbistatin group (n = 5), TNF-α group (n = 5), and TNF-α and blebbistatin group (n = 6). Representative data from two experiments are shown. *P < 0.05, **P < 0.01; measured using a Tukey’s test followed by two-way ANOVA.

Pro-inflammatory stimulation induces stress fiber formation in HUVECs via actin rearrangement^40, 41^. After TNF-α stimulation for 4 hours, rigid fibrillate structures were observed in the TNF-α-stimulated HUVECs associated with endothelial cellular stiffening (Fig. 1A). To study the effect of cytoskeletal rearrangement on endothelial cellular stiffness, we investigated the relationship between rigid fibrillate structure and actin rearrangement by using the Lifeact-enhanced green fluorescent protein (EGFP) fusion protein expressed in HUVECs. Cellular stiffness at the sites of actin accumulation was higher than that at non-accumulated areas in HUVECs (Fig. 2A). In addition, blebbistatin, a selective inhibitor of myosin II, attenuated TNF-α-induced rigid fibrillate structures and endothelial cellular stiffening (Fig. 2B,C). Cytochalasin D, an actin polymerization inhibitor, also impaired the rigid fibrillate structures and cellular stiffening of HUVECs (Supplemental Fig. 2). These findings suggest that TNF-α-induced actin rearrangement is a major determinant of endothelial cellular stiffening.

### Cell-cell interactions contribute to endothelial cellular stiffening

**Figure 3:**
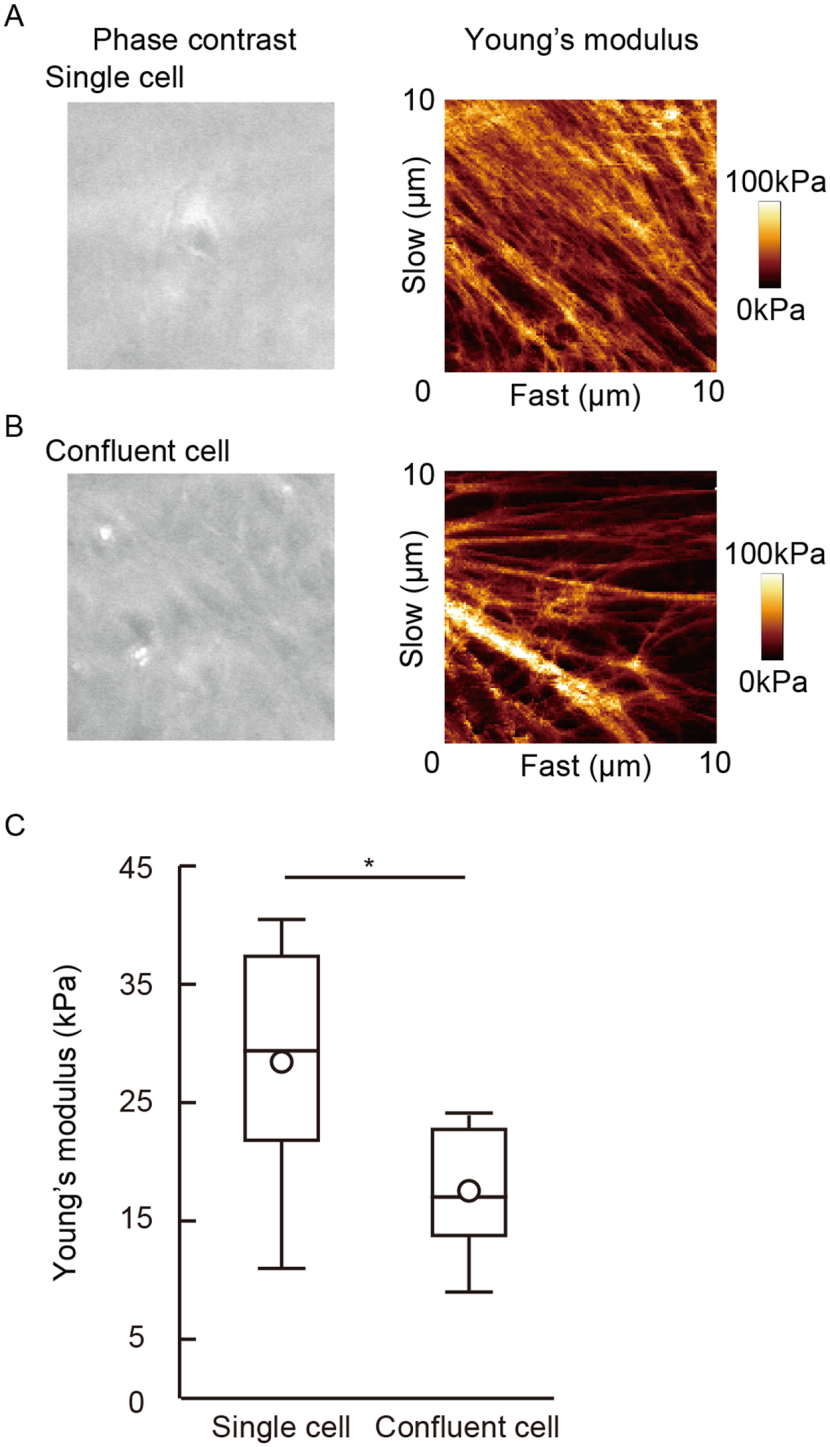
Cellular stiffness of a single cell and the confluent EC monolayer. Young’s modulus images of a single cell (**A**) and the confluent monolayer (**B**) were obtained. (**C**) The cellular stiffness of an individual cell (n = 6) and an adhered cell (n = 6) are shown. Representative data from three experiments are shown. *P < 0.05, **P < 0.01; measured using a Student t-test.

**Figure 4:**
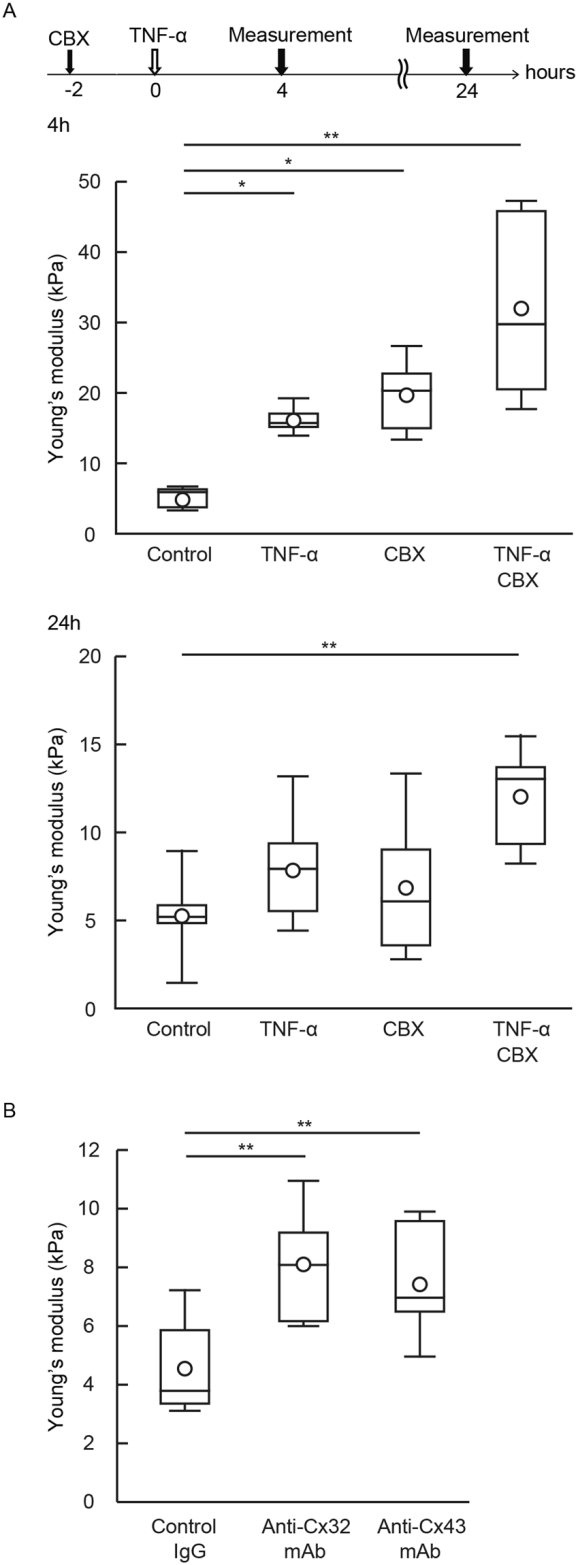
Gap junction inhibitor increased endothelial cellular stiffness. (**A**) HUVECs were treated with CBX for 2 hours and then stimulated with TNF-α for 4 hours. Young’s modulus of the control group (n = 5), CBX group (n = 5), TNF-α group (n = 6), and TNF-α and CBX group (n = 5) were determined at the indicated time points. Representative data from three experiments are shown. *P < 0.05, **P < 0.01; measured using a Tukey’s test followed by two-way ANOVA. (**B**) After being transferred with control IgG (n = 5), anti-Cx32 mAb (n = 7) and anti-Cx43 mAb (n = 8) for 6 hours, the stiffness of HUVECs was determined. Representative data from two experiments are shown. *P < 0.05, **P < 0.01; measured using a Tukey’s test followed by one-way ANOVA.

Cell-cell interactions influence the regulation of cellular stiffness and morphological changes^12^; however, the specific contributions of GJ-mediated cell-cell interactions to the regulation of cellular stiffness are as yet not fully understood. Consistent with the previous finding^12^, the stiffness of non-confluent HUVECs was higher than that of confluent HUVECs (Fig. 3), thereby supporting the contribution of cell-cell interactions to the regulation of cellular stiffness. As confluent cells are capable of signaling each other via GJs, this led us to examine the roles of GJs in regulating cellular stiffness. To interfere with the GJ functions, we treated confluent HUVECs with the GJ inhibitor carbenoxolone (CBX). CBX treatment increased endothelial cellular stiffness (Fig. 4A). The effects of individual applications of CBX or TNF-α in order to increase endothelial cellular stiffness were found to be transient in nature. The effects were seen at 4 hours or 6 hours but not at 24 hours (Fig. 4). By contrast, the effects of simultaneous application of CBX and TNF-α were found to be persistent, indeed remaining visible up to 24 hours. We confirmed that, like CBX, another inhibitor oleamide also increased endothelial cellular stiffness (data not shown). These inhibitor treatments blocked the GJ function of HUVECs, but did not alter the morphology of HUVECs (not shown).

Subsequently, we blocked GJ functionality via an alternative approach involving intracellular delivery of antibodies to the cytoplasmic domain of Cx^35, 36^. While the GJ inhibitors CBX and oleamide non-selectively inhibited Cxs^42, 43^, intracellularly transferred anti-Cxs monoclonal antibody (mAb) blocked Cx-specific GJ formation and function. HUVECs endogenously express Cx32, Cx37, Cx40, and Cx43, which form GJs^27^. We previously showed that Cx32 and Cx43 in HUVECs contribute more to the functional phenotypes of ECs than do Cx37 and Cx40^35, 36^. We therefore focused on Cx32 and Cx43, and intracellularly delivered anti-Cx32 mAb or anti-Cx43 mAb into HUVECs in order to block Cx32- and/or Cx43-mediated GJs (Fig. 4B). Both antibodies increased cellular stiffness compared with the use of control IgG. These results suggest that the GJs formed by Cx32 and/or Cx43 are important to the regulation of endothelial cellular stiffness.

### F-actin localization and focal adhesion formation in the GJ blockade of HUVECs

**Figure 5:**
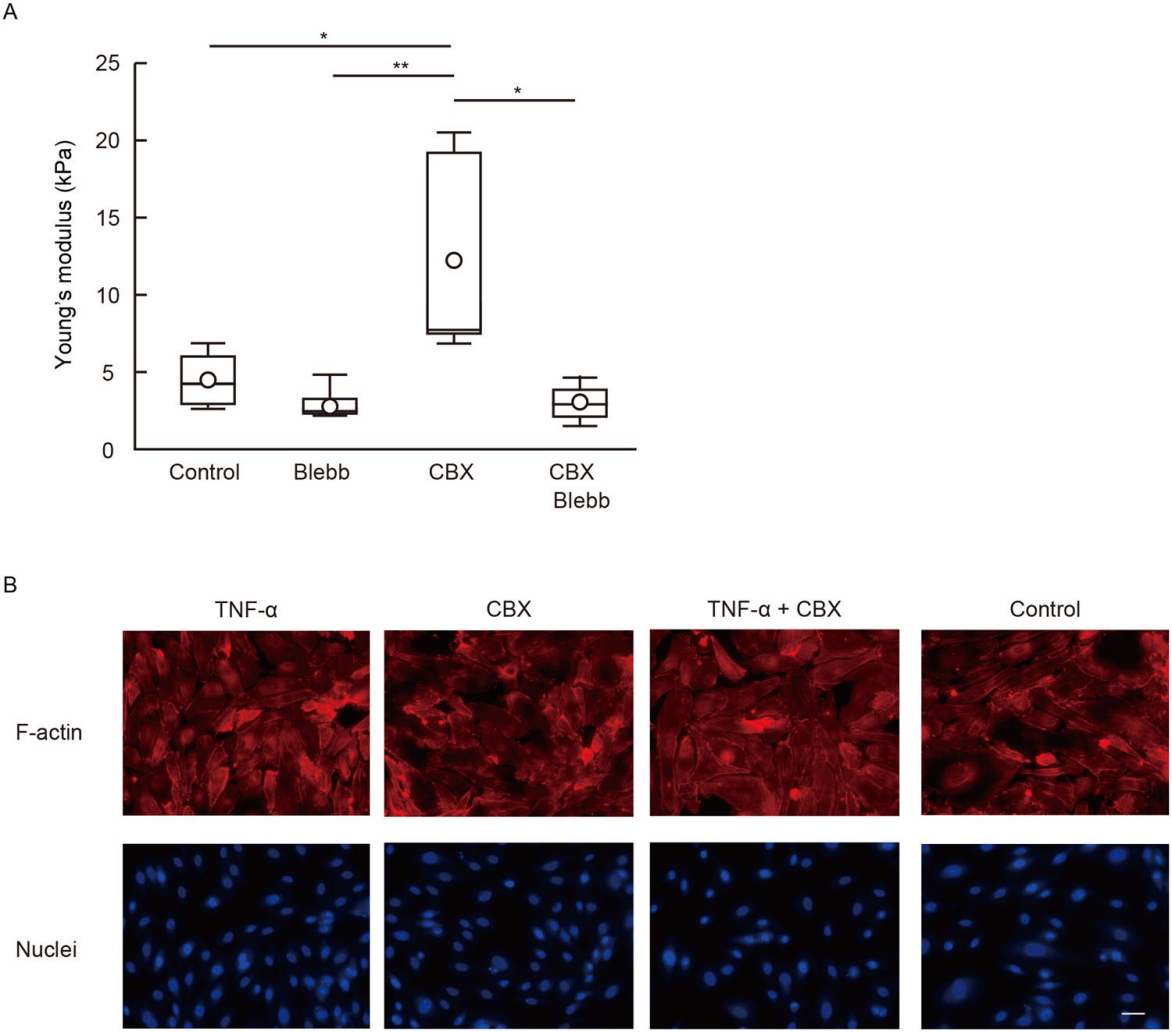
TNF-α and gap junction inhibitor induced actin rearrangement and focal adhesion formation. (**A**) HUVECs were treated with CBX and blebbistatin for 6 hours. The geometric means of the Young’s modulus of control group (n = 4), blebbistatin group (n = 5), CBX group (n = 5), and blebbistatin and CBX group (n = 5) were determined. Representative data from two experiments are shown. *P < 0.05, **P < 0.01; measured using a Tukey’s test followed by two-way ANOVA. (**B**) HUVECs were treated with CBX for 2 hours and then stimulated with TNF-α for 4 hours. F-actin (red) and nuclei (blue) were stained by rhodamine-phalloidin, and DAPI. Representative merged images from two experiments are shown. Scale bar showing 40 μm.

**Figure 6:**
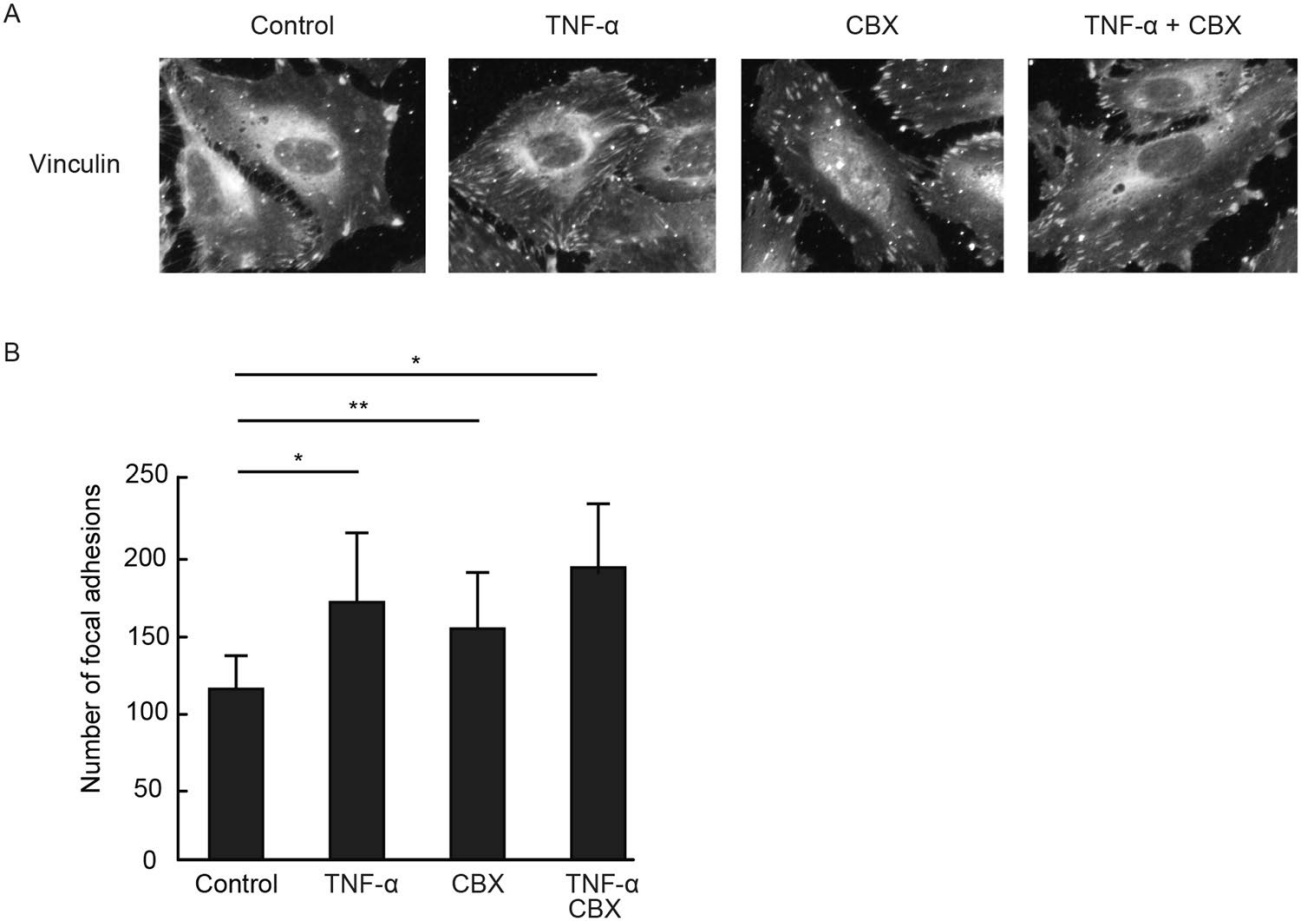
TNF-α and gap junction inhibitor enhanced vinculin accumulation and focal adhesion. (**A**) HUVECs were treated with CBX for 2 hours and then stimulated with TNF-α. Vinculin (green) were visualized by anti-vinculin mAb. Representative merged images from three experiments are shown. Scale bar showing 40 μm. (**B**) The focal adhesion number of each group (n = 6) was quantified using NIH ImageJ software. Data are expressed as the means ± SD. **P < 0.05, **P < 0.01 vs control group; measured using a Tukey’s test followed by two-way ANOVA.

We explored how GJ inhibition affected F-actin and focal adhesion. An individual application of TNF-α or CBX induced stress-fiber-like structures in HUVECs compared with untreated HUVECs (Fig. 5B), although total F-actin content, as assessed by flow cytometry, was not altered following TNF-α and/or CBX stimulation (Supplemental Fig. 3). Then we assessed the formation of focal adhesion in HUVECs by using vinculin staining (Fig. 6). The size and number of vinculin signals in HUVECs treated with TNF-α or CBX were greater compared with vehicle-treated control HUVECs. Cells simultaneously treated with TNF-α and CBX tended to increase the formation of focal adhesion compared with cells treated with TNF-α only. GJ inhibition appears to facilitate focal adhesion formations, thereby leading to cellular stiffening of HUVECs.

## Discussion

Several studies have suggested that the regulatory mechanism governing cellular stiffness involves a cell-to-substrate component^6, 7, 44^, as well as cell-to-cell interactions^22^. It has been reported that a non-confluent single cell promotes spreading and enhances cell-substrate interactions by integrin-dependent focal adhesion formation^12^. These cellular responses generate contraction forces in a single cell, thereby resulting in increasing cellular stiffness. In contrast to an individual cell, a cell in a confluent monolayer, which restricts the space available for cell spreading, establishes cell-cell junctions along with cell-substrate interactions^12^. As interactions with adjacent cells impair the generation of contraction forces via cell-to-substrate interactions, an individual cell is stiffer than a cell with a confluent monolayer. Furthermore, in a confluent monolayer, the weakening of cell-cell interactions by anti-vascular endothelial cadherin (VE-cadherin) antibody or low-dose cytochalasin B treatments augmented, in turn, cell-substrate interactions^12^, thereby leading to the stiffening of cells. Thus, the balance of adhesive strengths at the cell-substrate level (e.g., via integrins) and cell-cell (e.g, via VE-cadherin) interactions would be an important mechanism for determining cellular stiffness. This concept is consistent with our results that the weakening of Cx-mediated cell-cell interactions leads to an increase in focal adhesion formation, which is indicative of the augmented cell-substrate interactions associated with the stiffening of cells.

The link between Cxs with cytoskeletal proteins has been previously described. Chen and colleagues have reported that Cx43 associates with the F-actin cytoskeleton through zona occulden-1 (ZO-1), thus facilitating cell spreading and exploration during locomotion^45^. In addition, Cx proteins associate with other intracellular proteins, such as phosphatases and protein kinases, catenins, structural proteins, and microtubules. Cx32 interacts with Src, calmodulin, claudin, occludin, and β catenin^46, 47^, whereas Cx43 interacts with ZO-1, Src family members, protein kinases, phosphatases and others^48^. Moreover, other groups have found that GJs possibly regulate the cell migration and polarization associated with cytoskeletal rearrangement^49^. In our study, the data indicates that treatment with a GJ inhibitor enhances the formation of F-actin and focal adhesion. Our results suggest that GJs may modulate cellular stiffness through the actin rearrangement regulated by GJ-associated proteins.

In this study, we have shown that perturbation of GJ functions induced endothelial cellular stiffening and augmented TNF-α-mediated endothelial cellular stiffening partly by modulating cytoskeletal rearrangement and the formation of focal adhesion. While TNF-α induces EC activation and stiffening, it also, on the other hand, regulates Cx expression and the GJ function of ECs. It has been shown that TNF-α reduced mRNA and protein expression of Cx32, Cx37, and Cx40, and up-regulated Cx43 expression in cultured ECs^34, 50^. Furthermore, TNF-α did not alter Cxs mRNA and protein expression in cultured ECs after 4 hours, but TNF-α reduced endothelial GJ function^34^. Our results have shown that endothelial cellular stiffening occurs via the blockade of GJs with and without TNF-α stimulation, and that simultaneous application of a GJ inhibitor and TNF-α showed additive effects to augment endothelial cellular stiffness. It appears that a GJ blockade and TNF-α stimulation might act independently to induce endothelial cellular stiffening.

Whereas we used TNF-α-stimulated HUVECs as a model of inflamed endothelial cells, previous studies reported contrasting effects of TNF-α on endothelial cellular stiffness. Lee and colleagues described not only an increase in the mechanical stiffness of ECs after treatment with 10 ng/mL of TNF-α for 20 hours in the presence of serum, but also an increase of the density of F-actin filaments^2^. In contrast, Stroka and colleagues found that treatment with 10 ng/mL of TNF-α for 24 hours in the presence of serum decreased HUVEC stiffness, though it did induce F-actin rearrangements^7, 51^. Our results indicate that TNF-α transiently increases HUVEC stiffness after 4 hours of stimulation in serum-free media. Thereafter, HUVEC stiffness returns to the same stiffness level as those of non-stimulated normal ECs after 24 hours. As HUVECs have been shown to be more sensitive to TNF-α activity under serum-starved conditions, different culturing conditions might account for these contrasting results. Furthermore, both previous studies employed an AFM probe consisting of a 5 μm diameter particle at the edge of the cantilevers. In our AFM system, we utilized cantilevers with a tetrahedral type probe, in order to scan the heterogeneous distribution of cellular stiffness. Measurements of cellular stiffness are influenced by the force applied over the membrane, the velocity of the probe, and the depth distance of the probe. These different settings in the AFM measurements might also explain some of the discrepancies noted in the results.

Endothelial GJs play important roles in many vascular functions. The reduction in Cx37 and Cx40 expression, as well as the increase in Cx43 expression, have been reported following treatment of ECs using TNF-α^50^. Alterations in endothelial Cx expression patterns have been observed during the development of cardiovascular diseases^26, 52^. Cx37-deficient mice enhance the expression of those pro-inflammatory genes involved in advanced atherosclerosis^53^. In addition, Cx37 protects against early atherosclerotic lesion development by regulating monocyte adhesion^30^. The deletion of Cx40 in ECs promotes the development of atherosclerosis by leukocyte adhesion^29^. In heterogeneous Cx43 knock-out mice, atherosclerotic lesion formation was reduced^31^. We have previously shown not only that Cx32 expression in HUVECs is reduced after TNF-α stimulation, but also that attenuated Cx32 facilitates vascular inflammation and coagulation^27, 34^. Taken together, abnormal Cxs facilitate the development of atherosclerosis and vascular inflammation, both of which are based on endothelial dysfunction.

It is likely that leukocytes modulate cell adhesion, spreading, and migration depending on the degree of endothelial cellular stiffness^54, 55^. Neutrophils increased cell spreading on stiffer ECs, which served as a substrate for adherent neutrophils^7^. In addition, other studies, as well as our own, have shown the presence of heterogeneous stiffness gradients on the cell surface with fibrillate structures^51^. Thus, leukocytes may sense and respond to endothelial stiffness gradients from the moment of adhesion to the crawling phase of transmigration including durotaxis. The latter requires the sensing of substrate stiffness by adhesion molecule interactions^56^. Furthermore, endothelial GJs play an important role in monocyte recruitment. Specifically, reduced expression of Cx37 and Cx40 in ECs enhances monocyte adhesion and atherosclerotic plaque formation^30^. Cx40-mediated endothelial cell-cell communication positively regulates the expression of the ecto-enzyme CD73, which induces anti-inflammatory signaling via adenosine^29^ and prevents vascular cell adhesion molecule-1 (VCAM-1) expression. We also demonstrated that GJ-blocked ECs enhance intercellular adhesion molecule-1, VCAM1, and E-selectin expression after TNF-α stimulation^35^. Taken together, endothelial GJs may regulate leukocyte adhesion and transmigration through the modulation of both molecular expression patterns and the physical properties of ECs.

Arterial stiffening has been observed during atherosclerosis and is a cholesterol-independent risk factor for cardiovascular events^57–59^. Arterial stiffness is determined by the composition of matrix components, such as elastin and collagen, in the vascular wall^60^. In addition to the reconstitution of matrix components, the increased stiffness of vascular smooth muscle cells has also been associated with aging-related arterial stiffening^61^; however, the alteration and contribution of endothelial cellular stiffness remain unclear and has only been assumed from several *in vitro* and *in vivo* findings. Kothapalli showed the increased stiffness of the endothelial luminal surface in adventitia using an ApoE knockout atherosclerotic mouse model^8^. Schaefer and colleagues found *in vitro* that increased α-actinin-4 expression enhanced cellular stiffness of HUVECs, and *in vivo* that human and murine atherosclerotic plaques shows elevated levels of α-actinin-4 in ECs^9^, thereby suggesting that endothelial cellular stiffening could happen in atherosclerotic plaques *in vivo*. Our studies raise the possibility that aberrant endothelial GJs trigger endothelial cellular stiffening in the context of endothelial dysfunction.

## Methods

### Cell culture

Primary HUVECs, human aortic ECs, human pulmonary artery ECs, human lung microvascular ECs, and their culture media (EGM-2 BulletKit) were purchased from Lonza Japan (Tokyo, Japan). HUVECs were cultured in collagen-coated tissue-culture dishes (BD Biosciences, San Jose, CA) in an atmosphere containing 95% air and 5% CO_2_. All experiments were performed with cultured ECs during passages 3–5. Cultured HUVECs were starved overnight before treatment with TNF-α or thrombin. Recombinant TNF-α had an activity range of 1.0–100 × 10^6^ units/μg; 10 units/mL of TNF-α was smaller than 1 ng/mL. To stimulate HUVECs, cells in serum-free media were given either 10 units/mL of TNF-α or 1 units/mL of thrombin, and continued to be cultured. The stiffness measurements of stimulated HUVECs were performed at two time points: 4 and 24 hours after the addition of TNF-α or thrombin. These experiments were performed in triplicate and yielded similar results.

### Blockade of GJ function in HUVECs

In order to block GJ function, HUVECs were treated with 1 μM of CBX (Sigma, St. Louis, MO) for 2 hours and were then stimulated by TNF-α in the presence or absence of CBX. In our previous studies, 1 μM of CBX completely inhibited gap junction function of HUVECs^27^. Anti-Cx32 mAb (Thermo Fisher Scientific, Waltham, MA) and acti-Cx43 mAb (Thermo Fisher Scientific) were intracellularly transferred into HUVECs using PULSin protein delivery reagent (PolyPlus-transfection, New York, NY)^34^. Briefly, HUVECs were grown to a semi-confluent state with cell-cell contact occurring at the proliferation phase in collagen-coated 60 mm tissue culture dishes and were then washed with prewarmed PBS. Mixtures of 200 μL HEPES buffered saline (20 mM HEPES, 150 mM NaCl, pH 7.5), 2 μg of each antibody and 8 μL PULSin were incubated at room temperature for 15 min. HUVECs were incubated with 1800 μL Opti-MEM (Thermo Fisher Scientific) with 200 μL antibody/PULSin mixture at 37 °C for 4 hours. After washing with PBS, HUVECs were incubated with fresh serum-free medium. Purified mouse IgG (Sigma) from serum was used as a control IgG. After CBX treatment or antibody transfer for 2 hours, HUVECs were grown to a confluent state and were then stimulated with TNF-α (10 units/ml) for 4 hours and 24 hours. Subsequently, the stiffness of HUVECs that established the onset of cell-cell interactions next to adjacent cells was determined. These experiments ware performed at least two times.

### Visualization and inhibition of actin-cytoskeletal rearrangement

To stain filamentous actin in living HUVECs, the Lifeact- EGFP fusion protein expressing pcDNA4 vector (Thermo Fisher Scientific) was constructed. Lifeact-EGFP stained F-actin structures in cells without interfering in actin dynamics^62^. DNA fragments encoding Lifeact peptide and EGFP were amplified by PCR from pEGFP-C1 plasmid (Takara Bio, Shiga, Japan). PCR amplicon was ligated into pcDNA4 vector by In-Fusion HD Cloning Kit (Takara Bio). Lifeact-EGFP fusion protein expressing pcDNA4 vector was transfected to HUVECs by using lipofectamin2000 (Thermo Fisher Scientific). HUVECs were grown to 70% confluence in collagen-coated 60-mm tissue-culture dishes and washed with pre-warmed PBS. The transfection solution was prepared as follows, 190 μL Opti-MEM with 10 μL lipofectamin 2000 and 200 μL Opti-MEM with 3 μg of Lifeact- EGFP fusion protein expressing pcDNA4 vector were mixed and incubated at room temperature for 30 min. HUVECs were then incubated with a mixture of 1600 μL Opti-MEM and 400 μL in a transfection solution at 37 °C for 4 hours. After removing the transfection solution, HUVECs were incubated with fresh medium at 37 °C for 24 hours before use in experiments. After administration for 24 hours, Lifeact-EGFP positive HUVECs were observed under an Olympus IX71 fluorescence microscope and the cellular stiffness was measured.

HUVECs were treated with 10 μM of blebbistatin (Merck Millipore, Darmstadt, Germany) or 2 μM of Cytochalasin D, stimulated with TNF-α (10 units/ml) for 4 hours, and then the stiffness of the HUVECs was determined. This experiment was performed two times.

### AFM to measure cellular stiffness

Young’s modulus of live HUVEC monolayers was measured using the NanoWizard 3 AFM system (JPK Instruments AG, Berlin, Germany) with a cantilever and a tetrahedral type probe (BL-AC40TS-C2; Olympus, Japan)^63^. All force curves and scanning field images (10 μm × 10 μm) were recorded at a resolution of 128 × 128 pixels in Quantitative imaging (QI) mode at 37 °C. The maximum force and contact point determined by the vertical deflection of the cantilever was set to 0.5 nN, and the scan rates were automatically controlled by the Z length (1 μm), extension time (15 ms) and retraction time (15 ms). The force constant of the cantilever was 0.05–0.10 N/m. The force constant for each cantilever was calibrated using the thermal noise method^64^. The indentation of depth determined by the tip-sample separation at each set point was always smaller than 400 nm. The contact points were determined by calculating the point where the extending force curve crossed the baseline of vertical deflection. Young’s modulus of cells was examined using the force curve obtained by measuring the amount of cantilever deflection. The force curves were measured in the contact mode. The loading rate of the probe was 1 μm/s. The force curve during the extension of the Z-piezo was used to determine the Young’s modulus of the cell. The data were processed by curve-fitting with the Hertz contact model using JPK data processing software. The geometric mean of Young’s modulus was calculated from the acquired Young’s modulus at each point of the cell for a given condition. Fluorescence images and force curve images were fitted by JPK data processing software and direct overlay software (JPK).

### Fluorescent imaging of F-actin and vinculin in HUVECs

After stimulation with TNF-α and CBX, HUVECs were fixed by 4% paraformaldehyde, and then permeabilized by 0.05% tween in phosphate buffered saline. Actin were visualized using rhodamine-phalloidin (Thermo Fisher Scientific). Vinculin were stained with anti-human vinculin monoclonal antibody (Sigma) and Alexa488 conjugated anti-mouse IgG antibody. Nuclei were stained using 4′, 6-diamidino-2-phenylindole (DAPI). HUVECs were observed using Zeiss fluorescence microscopy. The number of vinculin spots was analyzed by using ImageJ software (US National Institutes of Health). This experiment was performed three times.

### Statistical analyses

Statistical tests between data pairs was carried out using a Student t-test, and differences between the groups were analyzed by using Tukey’s test followed by one-way ANOVA or two-way ANOVA; *P < 0.05, **P < 0.01 was considered statistically significant.

## Electronic supplementary material

SuppleFigures
