# Supplementary material for: Gap junction-mediated regulation of endothelial cellular stiffness
Source: Sci Rep. 2017 Jul 21;7:6134. doi: 10.1038/s41598-017-06463-x (PMC5522438; doi:10.1038/s41598-017-06463-x)
Supplement: Supplementary file 1 — SuppleFigures [file 41598_2017_6463_MOESM1_ESM.pdf]

Gap junction-mediated regulation of endothelial cellular stiffness

Takayuki Okamoto[1, 2, *], Eiji Kawamoto[2, 3], Yoshimi, Takagi[2], Nobuyuki Akita[4], Tatsuya Hayashi[5], Eun Jeong Park[2], Koji Suzuki[6], and Motomu Shimaoka[2, *]

[1] Department of Pharmacology, Faculty of Medicine, Shimane University, 89-1 Enya-cho, Izumo-city, Shimane 693-8501, Japan

[2] Department of Molecular Pathobiology and Cell Adhesion Biology, Mie University Graduate School of Medicine, 2-174 Edobashi, Tsu-city, Mie 514-8507, Japan

[3] Emergency and Critical Care Center, Mie University Hospital, 2-174 Edobashi, Tsu-city 514-8507, Japan

[4] Faculty of Medical Engineering, Suzuka University of Medical Science, 1001-1, Kishioka-cho, Suzuka-city, Mie 510-0293, Japan

[5] Department of Biochemistry, Mie Prefectural College of Nursing, 1-1-1 Yumegaoka, Tsu-city, Mie 514-0116, Japan

[6] Faculty of Pharmaceutical Science, Suzuka University of Medical Science, 3500-3, Minamitamagaki-cho, Suzuka-city, Mie 513-8679, Japan

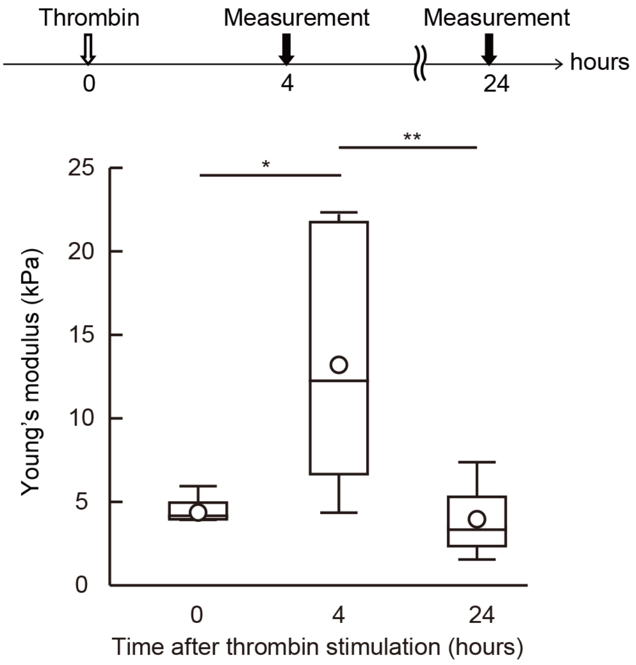

Supplementary Figure. 1. Pro-coagulant factor thrombin increased endothelial cellular stiffness. HUVECs were stimulated with thrombin and the force curve of the living cells was measured at the indicated time. The geometric means of Young's modulus of HUVECs was determined after thrombin stimulation for 0 (n=4), 4 (n=6), and 24 hours (n=8). *$P < 0.05$; **$P < 0.01$; measured using a Tukey's test. Representative data from three experiments are shown.

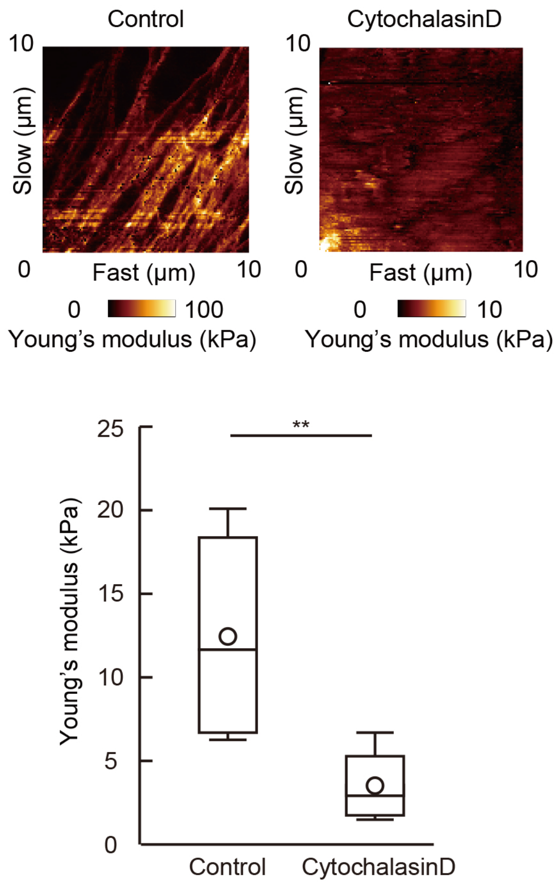

Supplementary Figure. 2. The heterogeneity of the cellular stiffness is dependent upon the cytoskeletal rearrangement. HUVECs were treated with 2μM of cytochalasin D for 1 hour. A Young's modulus of HUVECs was determined by AFM. Representative Young's modulus images of HUVECs were reconstructed. *P< 0.05; **P< 0.01; measured using a Student t-test. Representative data from three experiments are shown.

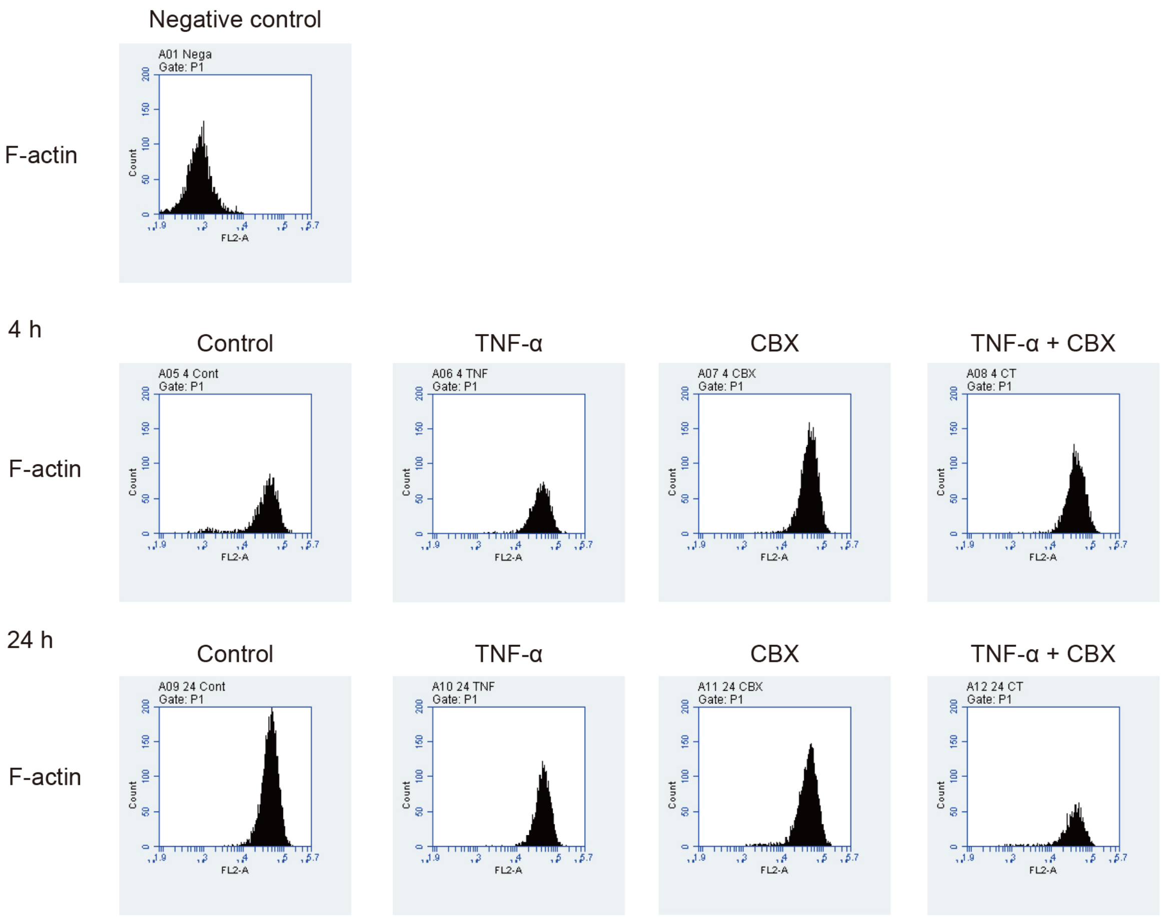

Supplementary Figure. 3. The amount of F-actin in HUVECs was quantified by flow cytometry. After TNF-α and/or CBX stimulation, HUVECs were fixed with 4% paraformaldehyde and stained by rhodamine-phalloidin. Samples were evaluated by a BD Biosciences Accuri C6 Personal Flow Cytometer. Fluorescence intensity of total F-actin content was identified for all samples. Ten thousand events were acquired for each sample. Results are displayed as histograms. Representative data from three experiments are shown.
